# An Eye on Survival: Association Between Initial Optic Nerve Sheath Diameter and 30-Day Mortality in Patients With Traumatic Brain Injury

**DOI:** 10.7759/cureus.114013

**Published:** 2026-08-05

**Authors:** Anagha C, Gomathi Karmegam, Satish Raja M C, Pavithra R, Shalini K, Harsha Vardhanan T, Sahasyaa Adalarasan

**Affiliations:** 1 Emergency Medicine, Madras Medical College, Chennai, IND; 2 Medicine, Madras Medical College, Chennai, IND

**Keywords:** intracranial pressure, mortality prediction, ocular ultrasonography, optic nerve sheath diameter, point-of-care ultrasound, prognostic biomarkers, traumatic brain injury

## Abstract

Background

Traumatic brain injury (TBI) is a major cause of morbidity and mortality worldwide, necessitating early identification of high-risk patients for timely management. Elevated intracranial pressure (ICP) is associated with poor outcomes in TBI, and optic nerve sheath diameter (ONSD), measured by bedside ultrasonography, has emerged as a useful noninvasive marker of elevated ICP. This study aimed to evaluate the association between initial ONSD measurement and 30-day mortality among patients with TBI.

Methods

A prospective observational study was conducted over one year in the Department of Emergency Medicine at Rajiv Gandhi Government General Hospital, Chennai, India. Sixty adult patients with clinically and radiologically confirmed TBI were included in the study. Patients with pre-existing intracranial or ocular pathology, direct ocular trauma, or delayed presentation beyond 48 hours were excluded. ONSD was measured in both eyes using bedside ultrasonography (MINDRAY system; Mindray Medical International Ltd., Shenzhen, China), and the mean value was recorded. Patients were followed for 30 days, and outcomes, including mortality, ICU stay, and duration of hospital stay, were documented. Statistical analysis included the chi-square test, independent t-test, analysis of variance (ANOVA), and receiver operating characteristic (ROC) curve analysis.

Results

The mean ONSD among study participants was 4.16 ± 0.70 mm. Fourteen patients (23.3%) died within 30 days, while 46 patients (76.7%) survived and were discharged. Mean ONSD was significantly higher among patients who expired compared to survivors (4.52 mm vs. 4.05 mm; 95% CI, p = 0.027). ROC analysis demonstrated an area under the curve (AUC) of 0.682 (95% CI: 0.549-0.796). An ONSD cut-off value >3.9 mm yielded a sensitivity of 78.57% and a specificity of 64.35% for identifying patients who experienced 30-day mortality. Increased ONSD was also significantly associated with lower Glasgow Coma Scale (GCS) scores, prolonged ICU stay, and longer hospital stay (95% CI, p = 0.001).

Conclusion

Initial ONSD measurement was significantly associated with injury severity and 30-day mortality in patients with TBI, supporting its potential role as an adjunct bedside marker for early risk stratification. Elevated ONSD was significantly associated with poorer neurological status and prolonged hospitalization, suggesting that bedside ONSD ultrasonography may serve as a useful non-invasive prognostic tool in emergency settings.

## Introduction

Traumatic brain injury (TBI) is a major cause of morbidity and mortality worldwide and constitutes a significant proportion of emergency department admissions following polytrauma [1]. Increased intracranial pressure (ICP) is one of the most serious complications of TBI and is associated with poor neurological outcomes, brain herniation, and death if not identified and treated early [1].

The optic nerve sheath is anatomically continuous with the intracranial subarachnoid space and is surrounded by cerebrospinal fluid [2]. As ICP rises, cerebrospinal fluid accumulation leads to distension of the optic nerve sheath, increasing optic nerve sheath diameter (ONSD). Recent studies have demonstrated a significant correlation between ONSD and elevated ICP, making ONSD a promising non-invasive surrogate marker for intracranial hypertension [3-5]. ONSD can be measured using ultrasonography, CT, or MRI; among these, bedside ultrasonography has gained importance due to its rapidity, portability, repeatability, and lack of ionising radiation. Additionally, bedside ultrasound-guided ONSD measurement can be performed quickly in the emergency department without delaying resuscitative measures [2,4].

At Rajiv Gandhi Government General Hospital, TBI forms a major component of polytrauma cases presenting to the emergency department. Common injuries include subdural haemorrhage, extradural haemorrhage, subarachnoid haemorrhage, cerebral contusions, diffuse cerebral oedema, and diffuse axonal injury. Early assessment of injury severity and mortality risk in patients with TBI is essential for appropriate triage and management. Therefore, this study was undertaken to evaluate the association between initial bedside ultrasonographic ONSD measurement and 30-day mortality in patients with TBI, as well as its relationship with clinical outcomes such as ICU stay and duration of hospitalisation.

## Materials and methods

This prospective, single-centre study was conducted in the Department of Emergency Medicine at Rajiv Gandhi Government General Hospital, Chennai, India, over one year from June 2025 to December 2025 to evaluate the association between initial ONSD measurement and 30-day mortality in patients with TBI. Adult patients aged 18-65 years presenting to the emergency department within 48 hours of head trauma and providing informed consent, either directly or through legally authorised representatives, were included in the study. Patients presenting beyond 48 hours of injury, those with major thoracoabdominal trauma (hemoperitoneum, massive haemothorax, or tension pneumothorax), pre-existing intracranial pathology, previous TBI, ocular pathology, or direct ocular trauma were excluded. Consecutive sampling was employed, and a total of 60 patients were enrolled. The sample size was calculated based on the study by Avci Ozbalik et al. [6], assuming an expected proportion of 91%, a confidence level of 95% (Z = 1.96), and an absolute precision of 7.5%, using the formula $n=\frac{Z^2p\left(1-p\right)}{d^2}$, yielding a minimum sample size of 55.94, which was rounded off to 60 participants.

Following initial stabilisation and resuscitation according to Advanced Trauma Life Support (ATLS) protocols [7], all patients underwent detailed clinical assessment and non-contrast CT of the brain to confirm TBI and document associated intracranial lesions. Bedside ultrasonographic measurement of ONSD was performed during the initial emergency department assessment after haemodynamic stabilisation using a MINDRAY ultrasound system (Mindray Medical International Ltd., Shenzhen, China) equipped with a high-frequency (7-15 MHz) linear array transducer. Patients were examined in the supine position with the head elevated to 30°. After application of sterile ultrasound gel over the closed eyelid, the probe was gently placed in the transverse plane, ensuring minimal pressure on the globe. The optic nerve sheath was identified in the plane containing the anterior chamber, lens, posterior chamber, and optic disc, and ONSD was measured 3 mm posterior to the globe, where the optic nerve sheath is most reliably assessed [8]. Two measurements were obtained from each eye, and the average of the four readings was used for analysis to improve measurement precision and minimise observer-related variability. All examinations were performed using a standardised scanning protocol.

Patients were followed throughout their hospital stay, and demographic, clinical, radiological, and outcome data were recorded. Neurological status was assessed using the Glasgow Coma Scale (GCS), an open-access clinical assessment tool developed by Teasdale and Jennett [9]. No permission was required for its use. The primary outcome measure was 30-day mortality, determined from hospital records and follow-up data. Statistical analysis was performed using IBM SPSS Statistics for Windows, Version 24 (Released 2016; IBM Corp., Armonk, NY, USA). Continuous variables were expressed as mean ± standard deviation (SD), whereas categorical variables were expressed as frequencies and percentages. Associations between categorical variables were analysed using the chi-square test. Comparisons of continuous variables between two independent groups were performed using the independent samples t-test. Comparisons across more than two groups were performed using one-way analysis of variance (ANOVA). Receiver operating characteristic (ROC) curve analysis was performed to evaluate the discriminatory performance of ONSD and determine the optimal cut-off value for distinguishing patients who died within 30 days from those who survived. The area under the curve (AUC), sensitivity, specificity, positive and negative likelihood ratios, and Youden index were calculated.

## Results

A total of 60 patients with TBI presenting to the emergency department were enrolled in this prospective observational study. Bedside ultrasonographic measurement of ONSD was performed in all eligible patients, and they were followed up for assessment of 30-day mortality and other clinical outcomes.

The study population had a mean age of 42.82 ± 15.15 years, with the majority of patients belonging to the 41-50 years age group (14/60, 23.3%). Male predominance was observed, accounting for 44 patients (73.3%) of the study population. The mean ONSD among the participants was 4.16 ± 0.70 mm. Most patients had no significant comorbid illnesses, observed in 36 patients (60.0%), while hypertension and type 2 diabetes mellitus were the most common comorbid conditions. The mean GCS score on presentation was 10.10 ± 4.37. Surgical intervention was required in 17 patients (28.3%) during the course of management. The baseline demographic and clinical characteristics are summarised in Table 1.

**Table 1:** Demographic data

S. no.	Demographics	Data
1	Age	42.8 ± 15.15 Years
2	Male	44 (73.3%)
3	Female	16 (26.7%)
4	Mean Optic Nerve Sheath Diameter	4.16 ± 0.70 mm
5	Mean Glasgow Coma Scale Score (GCS)	10.10 ± 4.37
6	Patients with comorbidities	24 (40%)
7	Patients who underwent surgical intervention	17 (28.3%)

Among the 60 study participants, 35 (58.3%) required an ICU stay of one to seven days, whereas 19 (31.7%) did not require ICU admission and six (10.0%) remained in the ICU for more than seven days. Regarding hospital stay, 11 patients (18.3%) were hospitalised for less than three days, 41 (68.3%) for 3-10 days, and eight (13.3%) for more than 10 days. Patients with hospital stays of less than three days had the highest mortality rate (6/11, 54.5%), indicating that shorter hospitalisation in this subgroup primarily reflected early mortality rather than rapid recovery. Overall, 46 patients (76.7%) were discharged, while 14 (23.3%) died during the study period.

Independent samples t-test demonstrated no statistically significant association between ONSD and age, gender, or comorbidities (p > 0.05). Surgical intervention was required in a minority of cases, and patients who underwent intervention had a significantly higher ONSD compared with those who did not (independent samples t-test, p = 0.001). Higher ONSD was also significantly associated with poorer clinical status and adverse outcomes. One-way ANOVA showed that patients with severe GCS scores (≤8) demonstrated the highest mean ONSD (4.58 ± 0.77 mm), compared with those with moderate (9-12) and mild (13-15) GCS scores (p = 0.001). Similarly, one-way ANOVA revealed that patients requiring ICU stay for more than seven days had markedly elevated ONSD values (5.32 ± 0.37 mm, p = 0.001). Increased ONSD was also observed among patients with prolonged hospital stay, while an independent samples t-test showed that patients who died had a significantly higher mean ONSD than those who were discharged (4.53 ± 0.78 mm vs. 4.05 ± 0.65 mm, p = 0.027). These findings suggest that elevated ONSD correlates with greater neurological severity and poorer clinical outcomes in patients with TBI.

The association between ONSD and clinical variables is shown in Tables 2-4. Mean ONSD increased significantly with worsening neurological status, prolonged ICU stay, and prolonged hospital stay. Patients with severe TBI (GCS ≤8) demonstrated the highest mean ONSD values. Similarly, patients requiring ICU stay for more than seven days and those with prolonged hospital stay exhibited significantly higher ONSD measurements.

**Table 2:** Association of ONSD with Glasgow Coma Scale (GCS) among study participants ONSD values are presented as mean ± standard deviation. Comparisons were performed using one-way ANOVA. *p < 0.05, indicating statistical significance. ONSD: optic nerve sheath diameter; ANOVA: analysis of variance

GCS category	Mean ONSD (mm) ± SD	Statistical test	p-value
13-15	3.76 ± 0.45	ANOVA (F = 9.73)	0.001*
9-12	4.17 ± 0.22	ANOVA (F = 9.73)	0.001*
≤8	4.58 ± 0.77	ANOVA (F = 9.73)	0.001*

**Table 3:** Association of ONSD with duration of ICU stay among study participants ONSD values are presented as mean ± standard deviation. Comparisons were performed using one-way ANOVA. *p < 0.05, indicating statistical significance. ONSD: optic nerve sheath diameter; ANOVA: analysis of variance

Duration of ICU stay	Mean ONSD (mm) ± SD	Statistical test	p-value
0 days	3.92 ± 0.44	ANOVA (F = 3.69)	0.001*
1-7 days	4.10 ± 0.68	ANOVA (F = 3.69)	0.001*
>7 days	5.32 ± 0.37	ANOVA (F = 3.69)	0.001*

**Table 4:** Association of ONSD with duration of hospital stay among study participants ONSD values are presented as mean ± standard deviation. Comparisons were performed using one-way ANOVA. *p < 0.05, indicating statistical significance. ONSD: optic nerve sheath diameter; ANOVA: analysis of variance

Duration of hospital stay	Mean ONSD (mm) ± SD	Statistical test	p-value
<3 days	4.53 ± 0.74	ANOVA (F = 3.84)	0.001*
3-10 days	3.98 ± 0.58	ANOVA (F = 3.84)	0.001*
>10 days	4.62 ± 0.88	ANOVA (F = 3.84)	0.001*

The association between ONSD and clinical outcome is shown in Table 5. Patients who died had a significantly higher mean ONSD compared with those who were discharged (4.53 ± 0.78 mm vs. 4.05 ± 0.65 mm; independent samples t-test, p = 0.027). These findings suggest that elevated ONSD is associated with increased mortality in patients with TBI.

**Table 5:** Association of ONSD with clinical outcome ONSD values are expressed as mean ± standard deviation. Comparison between groups was performed using independent samples t-test. *p < 0.05, indicating statistical significance. ONSD: optic nerve sheath diameter

Outcome	Mean ONSD (mm) ± SD	Statistical test	p-value
Discharged	4.05 ± 0.65	Independent samples t-test (t = 2.27)	0.027*
Death	4.53 ± 0.78	Independent samples t-test (t = 2.27)	0.027*

**Figure 1:**
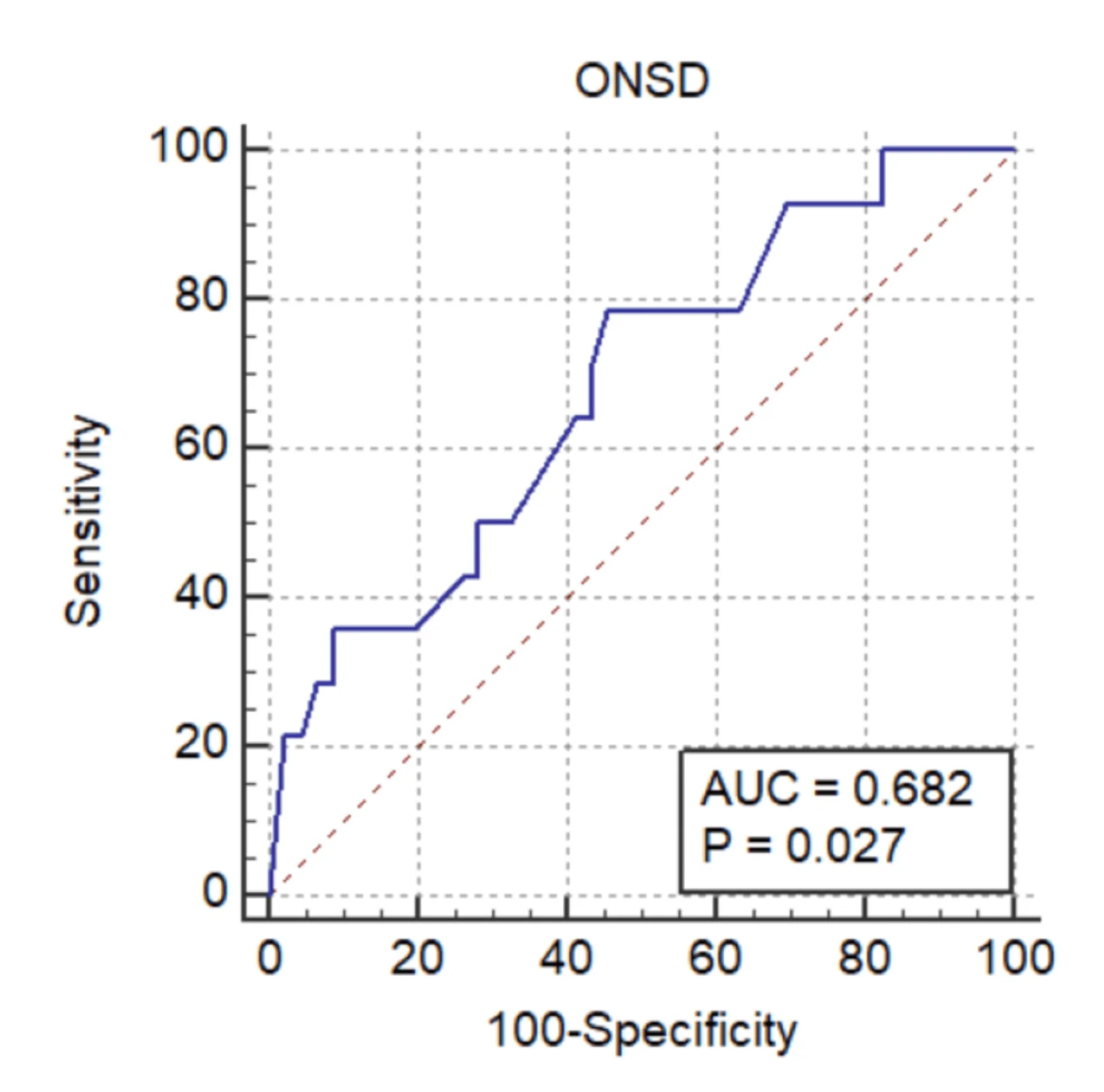
ROC curve of ONSD for discrimination of 30-day mortality among patients with traumatic brain injury ONSD: optic nerve sheath diameter; ROC: receiver operating characteristic; AUC: area under the curve

ROC curve analysis was performed to evaluate the discriminatory performance of ONSD for 30-day mortality among patients with TBI. The analysis demonstrated an AUC of 0.682 (95% CI: 0.549-0.796), indicating moderate discriminatory ability. An optimal ONSD cut-off value of >3.9 mm was identified using the Youden index. At this threshold, ONSD demonstrated a sensitivity of 78.57% (95% CI: 49.2%-95.3%) and a specificity of 64.35% (95% CI: 39.0%-69.1%) for distinguishing patients who died within 30 days from those who survived. The positive likelihood ratio was 1.72, whereas the negative likelihood ratio was 0.39. The results of ROC analysis are summarised in Table 6, and the ROC curve is illustrated in Figure 1.

**Table 6:** Diagnostic performance of ONSD for discrimination of 30-day mortality ONSD: optic nerve sheath diameter; ROC: receiver operating characteristic; AUC: area under the curve

Parameter	Value
Optimal ONSD cut-off (mm)	>3.9 (95% CI: 3.3-4.8)
Area under the curve (AUC)	0.682 (95% CI: 0.549-0.796)
Youden index	0.329
Sensitivity	78.57% (95% CI: 49.2%-95.3%)
Specificity	64.35% (95% CI: 39.0%-69.1%)
Positive likelihood ratio	1.72
Negative likelihood ratio	0.39

Overall, higher ONSD values were associated with poorer clinical outcomes, including lower GCS scores, prolonged ICU and hospital stay, increased requirement for surgical intervention, and higher mortality. These findings support the utility of bedside ultrasonographic ONSD measurement as a non-invasive adjunct for early risk stratification and assessment of injury severity in patients with TBI.

## Discussion

TBI is a major cause of mortality and long-term neurological disability worldwide, particularly among young adults and victims of road traffic accidents [1,10]. The clinical course of TBI is highly variable, ranging from mild transient symptoms to severe neurological deterioration and death [11,12]. Even patients with similar injury patterns may demonstrate markedly different outcomes, making early prognostication difficult in emergency and critical care settings. Since secondary brain injury caused by cerebral oedema, hypoxia, hypotension, and raised ICP can significantly worsen outcomes, rapid identification of high-risk patients is crucial for timely intervention, ICU prioritisation, neurosurgical referral, and family counselling [13].

Assessment of neurological status is commonly performed using the GCS [9,14] and even the Extended GCS (EGCS) [15]. Non-contrast CT with Rotterdam CT scoring is widely used for evaluating severity and prognosis in acute head injury [1]. However, GCS assessment may be unreliable in sedated, intubated, or paralysed patients, and invasive ICP monitoring techniques such as intraventricular catheterisation and intraparenchymal probes are associated with complications, including haemorrhage and infection [3]. Conventional prognostic models such as IMPACT and CRASH provide objective risk stratification using clinical and radiological parameters, but complex calculations and variability across populations may limit their utility in rapidly evolving emergency settings [16-19]. These limitations have increased interest in rapid, bedside, non-invasive prognostic tools that can assist in the early identification of patients at risk of poor neurological outcomes and help guide immediate management decisions in TBI [12].

ONSD has emerged as a valuable non-invasive marker for assessing ICP and predicting outcomes in TBI [4]. The optic nerve sheath is anatomically continuous with the intracranial subarachnoid space; therefore, elevations in ICP are transmitted along the cerebrospinal fluid pathway, leading to expansion of the sheath surrounding the optic nerve [2,20]. Because increased ICP contributes to cerebral hypoperfusion, ischemia, and secondary brain injury, rapid bedside identification of intracranial hypertension is crucial in patients with acute TBI [20]. Unlike invasive ICP monitoring techniques, which may be associated with haemorrhage, infection, technical difficulty, and limited availability, ONSD measurement provides a rapid, repeatable, and easily accessible alternative for early neuromonitoring [17,19,21].

The present study demonstrated the significant prognostic value of ONSD in TBI. Increased ONSD values were associated with lower GCS scores, higher Rotterdam CT severity scores, abnormal neuroimaging findings, intracranial bleeding, and poorer clinical outcomes [2,4,16]. These findings support the concept that raised ICP is reflected by expansion of the optic nerve sheath due to its continuity with the intracranial subarachnoid space [20]. Our study further highlights ONSD as a rapid, bedside, and non-invasive prognostic tool in TBI. Patients with more severe neurological and radiological involvement demonstrated significantly higher ONSD measurements, suggesting its usefulness in early risk stratification and identification of high-risk patients. Since ONSD enlargement occurs rapidly following ICP elevation, it may provide early prognostic information in emergency and critical care settings [2,4].

The prognostic value of ONSD observed in the present study is consistent with earlier work showing that optic nerve sheath enlargement reflects raised ICP and worse neurological outcome. In a prospective observational study of 100 patients with severe TBI, Robba et al. measured ONSD at admission to the neurocritical care unit and found significant correlations between ONSD and mean ICP, duration of ICP above 20 mmHg, pressure reactivity index, and invasively measured ICP estimated through ONSD-based noninvasive ICP calculation. They also reported that higher ONSD values were associated with increased mortality, supporting its role as an early screening tool for high-risk TBI patients [22]. This aligns well with our findings, where higher initial ONSD was associated with lower GCS scores, longer ICU stay, and higher mortality, suggesting that a single bedside measurement at presentation can provide meaningful prognostic information.

Similar results have also been reported in other neurological conditions. Zhu et al. studied comatose patients with supratentorial lesions using CT-based ONSD and ONSD/ETD ratio measurements and found that both were significantly higher in patients with poor prognosis than in those with better outcomes; however, the ONSD/ETD ratio showed better predictive performance than ONSD alone [3]. Likewise, Chelly et al. observed that larger ONSD values after cardiac arrest were associated with poorer survival and worse neurological outcome, with ONSD remaining an independent predictor even after adjustment for other clinical factors [23]. Bhide et al. further highlighted that ONSD is a useful bedside marker in non-traumatic neurocritical conditions as well, although cutoff values vary across studies and clinical settings [24].

In our study, despite evaluating initial ONSD alone, the same overall pattern was seen, with increasing ONSD corresponding to worse clinical severity and poorer outcome, reinforcing its practical value as a rapid prognostic marker in emergency TBI assessment.

The present study has several limitations. As a single-centre study with a relatively small sample size, the findings may not be fully generalisable to the wider population. Although ONSD demonstrated significant associations with mortality and other adverse clinical outcomes, only 14 mortality events occurred; therefore, multivariable logistic regression was not performed to avoid overfitting and unstable estimates. Consequently, the present findings demonstrate an association between ONSD and mortality rather than an independent prognostic effect after adjustment for established clinical predictors. Furthermore, interobserver and intraobserver reliability of ONSD measurements was not formally assessed, which may have contributed to measurement variability. In addition, the study was not designed to compare the performance of ONSD with established multivariable prognostic models such as IMPACT and CRASH. Future multicentre prospective studies with larger cohorts, adequate event numbers, standardised ultrasonographic protocols, formal assessment of measurement reliability, multivariable analyses, and direct comparison with validated prognostic models are warranted to further define the clinical role of ONSD in TBI.

## Conclusions

This prospective observational study evaluated the utility of ONSD as a prognostic marker for 30-day mortality in patients with TBI presenting to the department of emergency medicine. Increased ONSD values were associated with lower GCS scores, prolonged ICU and hospital stay, and higher mortality, supporting its role as an indicator of severe brain injury and raised ICP. Statistical analysis demonstrated moderate predictive ability, with an AUC of 0.682, sensitivity of 78.57%, and specificity of 64.35% for predicting 30-day mortality.

The findings of this study highlight ONSD as a rapid, non-invasive, and easily measurable bedside tool that may aid in early risk stratification and prognostic assessment in TBI patients. However, its predictive value should be interpreted alongside other clinical and radiological parameters such as GCS, associated injuries, and comorbidities. Further multicentre studies with larger sample sizes and standardised protocols are required to validate its clinical applicability and establish reliable prognostic cutoff values.
